# Changing socio-economic and ethnic disparities in influenza/A/H1N1 infection early in the 2009 UK epidemic: a descriptive analysis

**DOI:** 10.1186/s12879-021-06936-5

**Published:** 2021-12-11

**Authors:** James D. Munday, Richard Pebody, Katherine E. Atkins, Albert Jan van Hoek

**Affiliations:** 1 Centre for Mathematical Modelling of Infectious Diseases, London School of Hygiene and Tropical Medicine, London, UK; 2 Department of Infectious Disease Epidemiology, Faculty of Epidemiology and Population Health, London School of Hygiene and Tropical Medicine, London, UK; 3 Influenza and Other Respiratory Viruses Section, National Infection Service Colindale, Public Health England, London, UK; 4 Centre for Global Health, Usher Institute, College of Medicine and Veterinary Medicine, University of Edinburgh, Edinburgh, UK; 5 National Institute for Public Health and the Environment (RIVM), Bilthoven, The Netherlands

## Abstract

**Background:**

Higher incidence of and risk of hospitalisation and death from Influenza A(H1N1)pdm09 during the 2009 pandemic was reported in ethnic minority groups in many high-income settings including in the United Kingdom (UK). Many of these studies rely on geographical and temporal aggregation of cases and can be difficult to interpret due to the spatial and temporal factors in outbreak spread. Further, it can be challenging to distinguish between disparities in health outcomes caused by variation in transmission risk or disease severity.

**Methods:**

We used anonymised laboratory confirmed and suspected case data, classified by ethnicity and deprivation status, to evaluate how disparities in risk between socio-economic and ethnic groups vary over the early stages of the 2009 Influenza A(H1N1)pdm09 epidemic in Birmingham and London, two key cities in the emergence of the UK epidemic. We evaluated the relative risk of infection in key ethnic minority groups and by national and city level deprivation rank.

**Results:**

We calculated higher incidence in more deprived areas and in people of South Asian ethnicity in both Birmingham and London, although the magnitude of these disparities reduced with time. The clearest disparities existed in school-aged children in Birmingham, where the most deprived fifth of the population was 2.8 times more likely to be infected than the most affluent fifth of the population.

**Conclusions:**

Our analysis shows that although disparities in reported cases were present in the early phase of the Influenza A(H1N1)pdm09 outbreak in both Birmingham and London, they vary substantially depending on the period over which they are measured. Further, the development of disparities suggest that clustering of social groups play a key part as the outbreak appears to move from one ethnic and socio-demographic group to another. Finally, high incidence and large disparities between children indicate that they may hold an important role in driving inequalities.

**Supplementary Information:**

The online version contains supplementary material available at 10.1186/s12879-021-06936-5.

## Background

Inequalities in infectious disease outcomes are currently at the fore of public health interest, particularly in high income settings, where individuals with lower socio-economic and minority ethnic status have been at higher risk of severe disease and death during the COVID-19 pandemic [1–3]. Variation in outcomes between social groups is not unique to COVID. For instance, during the 2009/10 influenza A(H1N1)pdm09 (pH1N1) pandemic many high-income countries reported a higher incidence of various Influenza associated disease outcomes in certain social and ethnic sub-groups compared to the rest of the population [4–12]. In the UK, observations of disparities were most evident during the first few months of the outbreak, in the cities of London [13] and Birmingham [14]. These disparities can be due to differences in risk of acquisition, or in risk to develop clinical symptoms or severe disease. Analysis of inequalities in the risk of influenza infection can be challenging. Clear measurement of disparity in risk of acquisition of infection requires accurate, detailed data on cases at the point of infection. Without such data, analyses rely on proxy measures of infection such as hospitalisation or mortality [5, 7, 10, 11], where reported rates can be influenced by many factors not associated with transmission. Furthermore, by aggregating data with low spatial resolution, apparent associations between risk and social factors can become exacerbated or diluted due to confounding from geographical variation in infection risk within regions.

In many cases, sufficiently detailed case data is most frequently available from the early stages of an outbreak. Chiefly because the small number of cases allows: closer surveillance and better case ascertainment, higher proportion of cases tested in a laboratory, and more detailed patient records to be kept. Analysis of data early in an outbreak presents additional challenges however. For example, high degree of localisation in early outbreaks and residential clustering of social and ethnic groups geographically may result in measured inequalities simply as a result of the location of index cases and subsequent initial spread. Assessing how disparities in risk change over time might provide additional insight into what is driving them.

During the first three months of the UK pH1N1 epidemic in 2009, Public Health England (PHE, previously the Health Protection Agency) rolled out an influenza antiviral delivery program, in an effort to contain the outbreak by slowing onward transmission through reducing viral load and symptoms through early treatment of infected individuals and preventing infection in close contacts [15–18]. As part of this effort, data was collected from all those who reported symptoms and received treatment. Some patients were swabbed and samples sent for laboratory testing. After three months the response and surveillance effort was consolidated into the National Pandemic Flu Service (NPFS), where case data became less detailed [19].

To identify consistent patterns in the emergence of inequalities and gain insight into how disparities may arise, we compared the local outbreaks of Influenza A H1N1 in two cities, London and Birmingham, which between them accounted for the majority and highest density of cases during the period corresponding to the data we analysed. To do this, we analysed the anonymised individual level data collected during the initial antiviral delivery program to perform a detailed analysis of the socio-economic and ethnic breakdown of incidence of infection [20]. In particular, we assessed the way in which disparities between socio-economic and ethnic groups developed over the course of the early phase of the outbreak.

## Methods

### Data overview

We accessed the data, collected as part of the initial containment operation by PHE, from the Fluzone database [20]. The data provides a detailed record of the initial phase of the outbreak across the England and Wales. The anonymised individual level PHE data included: age, date of symptom onset, date that the report arrived at the test centre, full postcode (residence). The data also included case status, detailing lab testing result if applicable (confirmed or test-negative), or if untested, whether the case was considered ‘possible’, ‘probable’, ‘suspected’ or had been ‘excluded’ at the time of data collection.

To ensure only possible or confirmed cases were analysed, we excluded cases that were coded as either test-negative or ‘excluded’. For the purpose of the analysis we required both detailed location and symptom onset time, for cases where no date of symptom onset was recorded but did include the date received at test centre we estimated the date of symptom onset using the mean time between symptom onset and time received at test centre, as calculated from cases which had both dates recorded. Cases with neither reported were not included.

To provide details of socio-economic status and ethnicity breakdown by area (Table 1), we used UK 2011 census data accessed via the Office for National Statistics (ONS) website. Using the postcode, we assigned each case a Lower Super Output Area (LSOA), which are small geographical regions defined by ONS, with populations of between 800 and 2000 residents. we then linked population data to each case using LSOA level aggregates of the following fields from the 2011 census and 2010 IMD rankings:

**Table 1:** Breakdown of Birmingham and London by census reported Ethnicity (2011) and National IMD decile (2010) (1 is the most deprived decile) and age (2011)

	London (5)	Birmingham (%)
Ethnicity^a^		
White	59.79	57.93
South Asian	16.97	25.43
Black	13.32	8.98
Chinese	1.52	1.18
Other	8.40	6.47
IMD Decile^b^		
1	8.65	38.82
2	18.22	15.89
3	17.42	10.33
4	13.04	9.73
5	10.48	10.27
6	9.23	6.68
7	7.69	2.10
8	6.95	3.01
9	5.56	1.45
10	2.77	1.72
Ages		
0–9	12.11	13.36
10–19	11.49	14.55
20–29	18.11	16.96
30–39	18.09	14.23
40–49	14.44	13.25
50–59	10.31	10.20
60–69	7.42	7.94
70 +	8.02	9.51

^a^Based on 2011 Census estimates

^b^Based on 2010 IMD rankings and 2011 Census population estimates

Age distribution: The number of residents of each age from 0 to 79 and the number of residents 80 and over.

Ethnic group: The number of people who identify as each of the 18 census defined ethnic groups. This data was also broken down by age, which allowed ethnic breakdown to be estimated for children (≤ 19 years) and adults (> 19 years) separately. Ethnic group is coded as shown in Tables 1 and 2.

**Table 2:** Ethnic Group returned by Onomap and the corresponding UK Census codes that were used for population relative population size

ONOMAP Ethnic Group	Census Ethnic Group
White	White: English/Welsh/Scottish/Northern Irish/British
	White: Irish
	White: Gypsy or Irish Traveller
	White: Other White
South Asian	Asian/Asian British: Indian
	Asian/Asian British: Pakistani
	Asian/Asian British: Bangladeshi
Chinese	Asian/Asian British: Chinese
Other Asian	Asian/Asian British: Other Asian
Black	Black/African/Caribbean/Black British: African
	Black/African/Caribbean/Black British: Caribbean
	Black/African/Caribbean/Black British: Other Black
Other/Unclassified	Other ethnic group: Arab
	Other ethnic group: Any other ethnic group
	Mixed/multiple ethnic group: White and Black Caribbean
	Mixed/multiple ethnic group: White and Black African
	Mixed/multiple ethnic group: White and Asian
	Mixed/multiple ethnic group: Other Mixed

Deprivation: National Index of Multiple Deprivation (IMD) rank. The rank of the LSOA out of 34,753 LSOAs in England and Wales based on the IMD, a deprivation measure, which captures multiple facets of deprivation including wealth, income, living conditions, quality of life and health outcomes [21].

To assess socio-economic status by relative national deprivation and relative local deprivation, we summarised the deprivation by assigning each LSOA a national decile (the decile (10% band) of the IMD rank in England and Wales). In addition, we identified the local quintile (the quintile (20% band) of the IMD rank in the local area (either Birmingham or London)) of the LSOA in which each case lived.

To classify the ethnic group of each individual case, ethnicity was assigned at an individual level using Onomap software [22], this software uses first and last name, prior to anonymisation. The ethnicity classification for this analysis was performed by PHE prior to the commencement of this analysis. Although the inferred ethnicities are based on UK census ethnic groups, the software is not as precise as the census tract presenting a set of broader groups. We aggregated census ethnic groups to reflect those in Onomap to provide relevant denominators (Table 2). Onomap has been validated in the past [22] and has previously been used for similar analysis of influenza related mortality [23]. A summary of this verification is included in the supplementary information.

### Estimating socio-economic breakdown of cases

To estimate the distribution of cases in London and Birmingham by socio-economic status, we calculated the incidence proportion per 100k in each ten-year age group for each national IMD decile. To estimate the distribution of cases by local relative deprivation, we calculated the same for each local IMD quintile, calculated as the quintile of deprivation rank in the Birmingham local authority and the Greater London Authority areas for each city as appropriate.

To summarise the distribution of cases as the outbreak progressed, we plotted Lorentz curves for each week of the outbreak, calculated from the cumulative incidence in each week. The Lorentz plot shows the cumulative proportion of cases by deprivation quintile (i.e. proportion of cases in the most deprived 20%, proportion of cases in the most deprived 40% etc.). An equal distribution of cases by socio-economic status would return a straight line where the proportion of cases increases by 20% per deprivation quintile; this is called ‘the equity line’. Deviation from this line indicates unequal distribution of cases. A Lorentz curve below the equity line indicates a disproportionate share of cases in more affluent areas, whereas a curve above the line indicates disproportionate share of cases in more deprived areas.

To express unequal distribution of cases by deprivation status in a single value, we calculated the deviation from equity, $D$, which is the sum of the difference between the Lorentz curve and the equity line at each quintile (or the area between the Lorentz curve and the equity line). Hence a $D$ of 3 would indicate that all the cases were in the most deprived quintile, a D of -3 would indicate that all the cases were in the most affluent quintile. A value of 0 indicates no deviation from equity and cases were distributed equally amongst areas of different deprivation status.

### Estimating ethnic breakdown of cases using onomap ethnicity linkage

To estimate the proportion of the population each ethnic group comprised, we aggregated the Census ethnic groups as detailed in Table 2. To assess the variation in ethnic distribution of cases over the course of the outbreak, we calculated the relative risk at each day of the outbreak based on the cumulative incidence up to that day. As Onomap had not able to attribute an ethnic group to every case we only included cases that were assigned as one of: white, South Asian, Other Asian, Chinese or Black. In line with this we only calculated the relative proportion of the population amongst only these groups as well. (i.e. proportion of the population that is white is taken as the white proportion of a subset of the total population that is either White, South Asian, Other Asian, Chinese or Black).

We calculated the relative risk of infection in each ethnic group, $R{R}_{et}$, as the ratio of the proportion of cases in each group, ${P}_{cases,et}$, and the proportion of the population each group comprise, ${P}_{pop,et}$.

$$R{R}_{et}=\frac{{P}_{cases,et}}{{P}_{pop,et}}.$$

This is the equivalent of the risk of infection relative to what would be expected in an equitable scenario. We chose not to estimate the risk relative to a particular ethnic group to avoid an ethnically normative narrative. To ensure the results were not impacted by varying ethnic breakdown by age, we repeated this analysis for the total population and separately for children (≤ 19 years) and adults (> 19 years).

All data analysis was undertaken within PHE data-secure network using Python 2.7 [24]. Public Health England (PHE) holds permissions under sections 251 of the 2006 NHS Act and the 2002 Health Service (Control of Patient Information) Regulations, to process patient information.

Confidence intervals were calculated from the standard error as demonstrated by Morris and Gardner [25] (Additional file 1).

## Results

### Overview of the outbreak

The data comprised 20,301 reported cases nationally over the period of investigation, 12,018 cases remained after excluding cases based on case status. There were 1920 with postcodes within Birmingham LSOAs and 3631 with London LSOAs, which also reported either date of symptom onset or date of arrival at the test centre. Of these 855 and 1199 were confirmed in a laboratory for Birmingham and London, respectively. The rest remained ‘suspect’ or ‘possible’. Finally, 1315 of 1920 casess and 2486 of 3631 cases had ethnic group successfully inferred using Onomap.

Evaluating the bias of missing data showed no strong correlation between missing postcode information and any variable used in the analysis (Additional file 1). There is evidence of a lower test-positivity rate for submitted for laboratory testing amongst white individuals than other ethnic groups, which suggests that a higher proportion of the un-confirmed cases (probable, possible or suspected) may be false reports (Additional file 1).

### Age distribution of cases

**Fig. 1:**
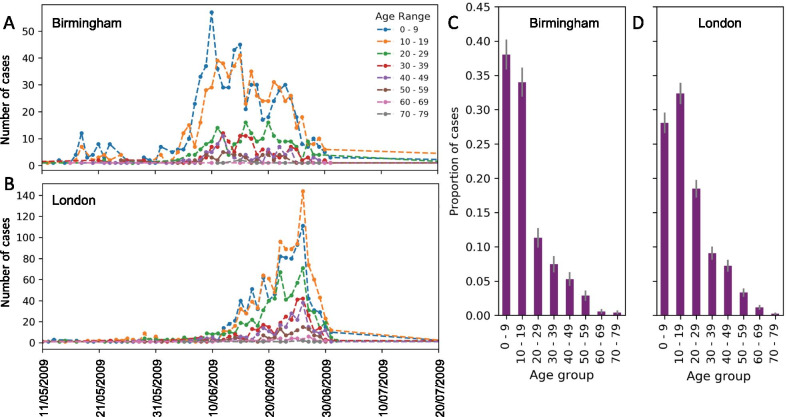
Age distribution of cases in Birmingham and London. Number of cases reported per day between 11th May and 27th July 2009 in (**A**) Birmingham and (**B**) London stratified by age group. Proportion of cases in each 10 year age group in (**C**) Birmingham and (**D**) London

In Birmingham 72% (70–4%, 95% CI) of cases were in children under the age of 19 years whereas in London this figure was lower with 60% (58–62%, 95% CI) of cases in this age group. In particular there was a higher proportion of cases reported in young adults, between 20 and 29 years of age, 18% (17–19%, 95% CI) in London, compared to 11% (10–12%, 95% CI) in Birmingham. The majority of cases in both outbreaks were reported in the same 10-week period from the 11th May to 27th July 2009 (day 130 to day 200 of the epidemic) (Fig. 1). Although there was a higher number of cases in London overall, there was a higher Incidence proportion in Birmingham.

### Cases by socio-economic status

**Fig. 2:**
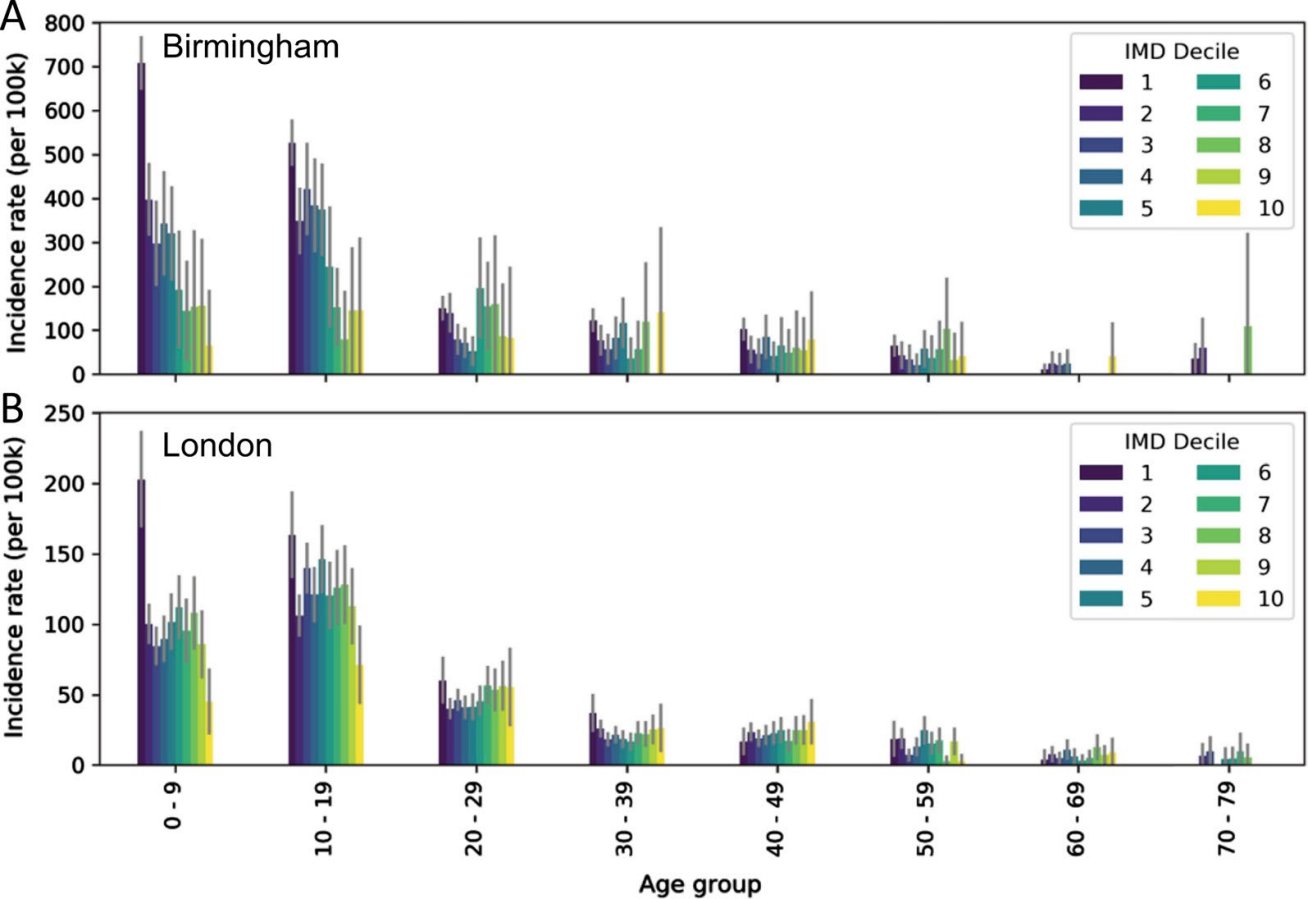
Incidence in each 10 year age group per national Index of Multiple Deprivation decile in (**A**) Birmingham and (**B**) London

**Fig. 3:**
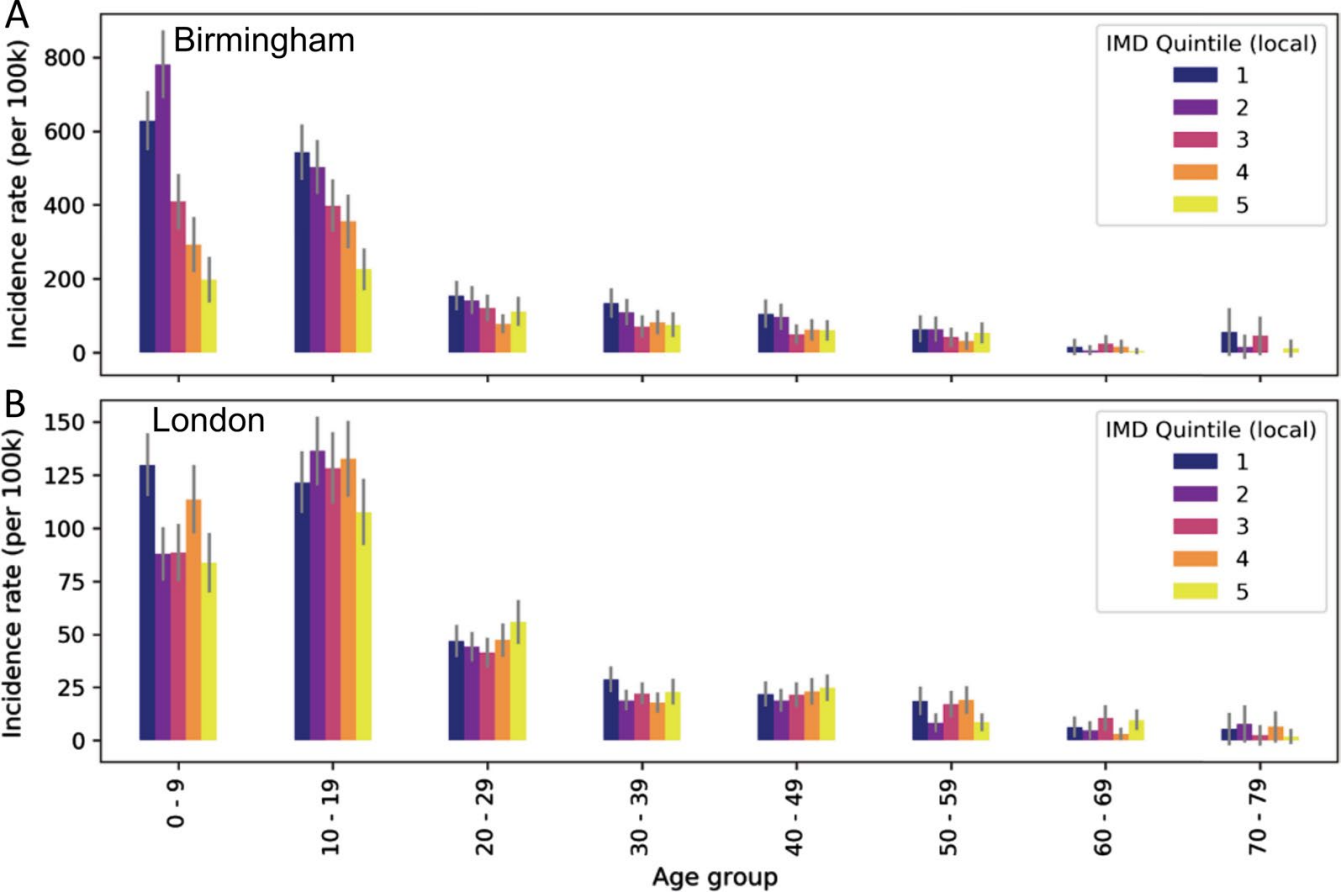
Incidence in each 10 year age group per local Index of Multiple Deprivation quintile in (**A**) Birmingham and (**B**) London

In Birmingham there was markedly higher incidence in children (0–19 years) in the lower national IMD deciles than the higher deciles, indicating higher incidence in more deprived areas of the city for this age-group (Fig. 2). This was also true for local IMD quintiles, where a reduced incidence was clear in more affluent quintiles (Fig. 3). Overall, incidence per 100 k in the most deprived 20% was 2.83 times higher than the most affluent 20%.

Conversely, in London, the difference in incidence proportion by deprivation was less clear than in Birmingham, using either national or local grouping of IMD rank. There was, however, substantially higher incidence proportion in the most deprived quantile than the most affluent quantile for 0–9 and 10–19 year olds when grouped based on national rank and for 0–9 year-olds when grouped locally. Overall, there was slightly higher incidence (1.37 times) in the most deprived 20% than the most affluent 20%.

The Lorentz curves for Birmingham of cumulative weekly incidence shows that the outbreak began in mostly deprived areas, with all cases in areas in the most deprived 40% and a deviation from equity value of 1.6, spreading to more affluent areas gradually, resulting in a deviation from equity of 0.6, and 60% of cases in the most deprived 40% of the population.

**Fig. 4:**
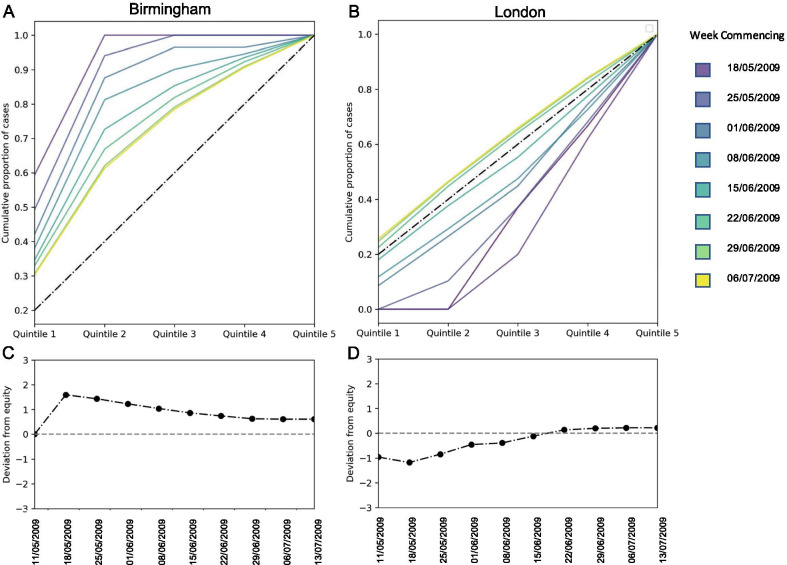
Disparities in incidence between local deprivation quintile over time as: Cumulative incidence by deprivation quintile in each week (Lorentz plot) in (**A**) Birmingham and (**B**) London; Deviation from equity for (**C**) Birmingham and (**D**) London

In contrast Lorentz curves for London indicate that the outbreak began in more affluent areas with a deviation from equity of − 1. The outbreak then progressed to infect more individuals from more deprived area eventually reaching a deviation from equity of 0.2 (Fig. 4).

### Cases by ethnic group

**Fig. 5:**
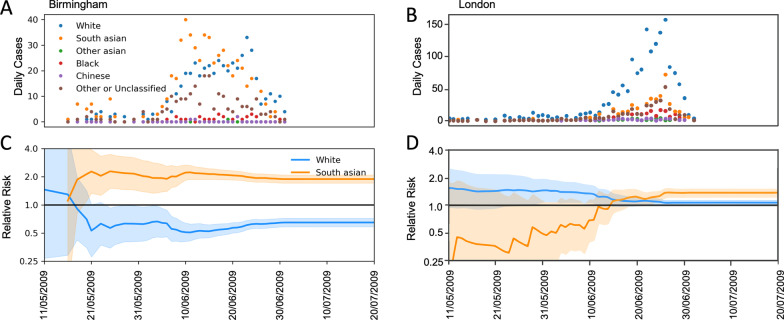
Breakdown of reported cases by ethnic group as determined by Onomap. Reported daily incidence of Influenza A(H1N1)pdm09 by ethnic group in (**A**) Birmingham and (**B**) London. Relative risk in White and South Asian ethnic groups in (**C**) Birmingham and (**D**) London; the solid lines show the calculated relative risk (compared to risk of the total population), the shaded areas show the corresponding 95% confidence limits

In Birmingham the majority of cases were in individuals identified by Onomap as White (37%) or South Asian (43%). The proportion of the population that was South Asian, however was substantially lower, which results in a relative risk of infection in South Asians of 1.89 (1.71–2.08, 95% CI) compared to White of 0.64 (0.58–0.72, 95% CI). The relative risk of infection based on the cumulative incidence at each day of the outbreak reveals that the outbreak began by infecting mostly white individuals. After 12th May, the outbreak either progressed or a new outbreak was introduced into the South Asian population, and then disproportionately affected this ethnic group from this point onwards. (Fig. 5).

In London, the majority of cases were in individuals identified as White by Onomap. South Asians made up 16% of all cases, but South Asians also make up a smaller proportion of the population than in Birmingham. The resulting relative risk in this group was 1.36 (1.22–1.51, 95% CI). By assessing the relative risk, based on cumulative incidence, at each day of the outbreak, it appears that the outbreak started in a largely white population. The proportion of cases in South Asians was lower than expected for much of the containment period, however there were only few cases and the confidence intervals included 1.0 (minimum RR was 0.24 (0.032–1.72, 95% CI). When incidence increased, a higher proportion of cases were in South Asian individuals, reaching a relative risk in this population of 1.36 (1.22–1.51, 95% CI), by the end of the data collection period (Fig. 5). The relative risks followed a similar trend in both adult and child age groups (children ≤ 19 years and adults > 19 years), the relative risk was slightly reduced when stratified by age, however the disparity remained clear (Additional file 1).

## Discussion

Disparities in incidence of pandemic influenza between ethnic groups have been reported in multiple high-income settings. Often the analyses which identify such disparities provide a single estimate of risk over a defined period of time or using data on particular health outcomes such as hospitalization or mortality, frequently using ecological analysis to infer relative risk between socio-economic and ethnic groups. By analysing individual level data of cases reported during the initial phase of the UK pH1N1 epidemic in two urban settings we found that disparities between socio-economic and ethnic groups were clear at the beginning of the local outbreaks in both cities, but much greater in Birmingham than London. Furthermore, the magnitude of the disparities between socio-economic and ethnic groups decreased over the first three months of the UK epidemic, tending towards equity throughout the containment period. Although the socio-economic and ethnic breakdown of cases at the start of the local outbreaks in Birmingham and London differed greatly, both appeared to reach an equilibrium with slightly higher incidence in South Asian population and more deprived areas.

Our analysis used individual level case data aggregated at high geographical resolution to evaluate how the outbreak progresses over time, which allows much clearer evaluation of how variation between groups changed over time. The high spatial aggregation of cases allowed us to better account for geographical variation in deprivation and ethnic group that analyses that evaluate inequalities at a lower resolution [13, 14].

We measured much clearer disparities by deprivation status in Birmingham than in London over the course of the data collection period, which were particularly clear amongst children. These finding corroborate previous analyses of inequalities over the same time period. A previous analysis [14] by Inglis et al. identified a disproportionate incidence in South Asians and more deprived regions in the West Midlands region overall (of which the city of Birmingham is an important part). Our analysis refines these findings by evaluating disparities at a finer spatial resolution and analysing how they change over time. Previous analysis, using a different dataset, of a similar period of the outbreak in London also identified a gradual transition of cases from more affluent early in the outbreak to more deprived communities [13]. Our analysis complements this by supporting the findings with a different dataset and evaluating disparities by ethnic group in this setting. A key benefit of our analysis is that by using a national dataset we were also able to make a direct comparison between the outbreaks in both cities.

Disparity in incidence by deprivation status is clearest in children (0–19 years), who also account for a large proportion of cases in both settings. Although previous analyses have demonstrated similar disparities amongst children in pandemic-related mortality [23], previous analyses of disparities between socio-economic and ethnic groups in infection data neglect age [14]. The proportion of cases in the 0–19-year-old age group increases as incidence increases overall. This suggests that: influenza transmission between children may have provided the basis for sustained transmission, and differences in transmission within this age group may drive the overall disparities observed. This highlights the potential role of outbreaks in particular schools in the way inequalities may emerge and change over the course of an influenza epidemic. Conversely, in both cities the relative risk of infection in South Asians was lower in children than adults. It’s important to note, however, that the proportion of cases in South Asians is higher in children than adults, but a higher proportion of children are South Asian overall resulting in a lower relative risk. This would suggest that in any epidemic driven by school-aged children, certain ethic minorities would be disproportionately affected, highlighting the importance of effective school-based surveillance strategies.

In both Birmingham and London, the distribution of cumulative cases by socio-economic status and ethnic group steadily tended towards equal distribution over the course of the containment phase. This characteristic may suggest that if an outbreak initiates in one particular socio-economic class it may persist in that group for a period of time before dispersing to another. Another important observation is that the outbreak in London appears to remain subdued, with low incidence for a prolonged period. Hence there is no evidence of sustained transmission within the population until 10th June, after which incidence increases. The increase in incidence coincides with an increase in the proportion of cases reported in more deprived areas. In Birmingham, high incidence occurred earlier in the outbreak and mostly in more deprived areas. Incidence then gradually increases in more affluent areas as well. In both the Birmingham and London, an increase in incidence coincided with an increase in the proportion of cases in areas with higher density of South Asians, in Birmingham this occurred around 12th May and in London around 10th June. In Birmingham the majority of cases classified by Onomap were South Asians. The coincidence of increased incidence and presence of cases in the most deprived quintiles—and South Asian populations could be driven by many and multiple phenomena. There are two important possible factors, which would be consistent with observations: firstly, sustained transmission may be more likely to start within more deprived regions and South Asian populations due to variation in surveillance and interaction with healthcare. Similar to recent findings from Zipfel et al. [26] in the United States, which suggest that lower visibility [27] and poor access to health care combine with isolation of particular groups on social networks to create inequalities in population risk regardless of individual level transmission risk. A second explanation could be that when sustained transmission occurs, transmission rates are generally higher in South Asian and deprived populations. Both of these factors would create inequalities in incidence of infection early on in an outbreak, the first due to higher incidence temporarily in that particular group due to seeding location, the second by transmission rate in a particular group leading to faster accumulation of cases and potentially higher overall incidence. Or indeed these factors could both be present, each compounding the other. The presence of inequalities in both setting suggest that if this is the case, a single property that these populations possess that drives this disparity may be present in both cities. Other explanations for this effect include a possibility that the high incidence in South Asians in Birmingham after 12th May sparked an outbreak in South Asians in London later through long-range social or familial contact links, leading to replication of the observed inequalities at this early stage, when cases were relatively few. There is also the possibility that the observed effects happened purely by chance for example if cases were disproportionately imported into South Asian communities. However, the relatively large number of cases in London before sustained transmission occurred suggests that seeding alone could not explain the observations, and that there were many opportunities for outbreaks in white communities earlier in the containment phase.

Interestingly, whereas we observed a clear relationship between incidence and deprivation status in children, the same relationship was not present in adults. This is curious as it might be expected that adults in a household with infected children would be at increased risk of infection. The analysis here offers no insight into why this phenomenon occurs, plausible contributing factors may be variation between social groups in household size or immunity due to prior infections. Another explanation could be that small outbreaks in mainly adult populations may have occurred in parallel, increasing the proportion of cases separately from the large outbreak amongst children. However, this finding does highlight the likely important role that deprivation can play in driving the early stages of an influenza pandemic, which has implications for control and prevention measures.

When analysing data collected as part of an initial outbreak response there are always some limitations that give way to potential biases. Firstly, some of the data collected was part of a contact tracing effort [20]. This creates the opportunity for biases to arise, as potential increased surveillance in certain populations may be compounded by active case finding amongst contacts in that population. Both of these may generate a higher case to infection ratio. There is evidence however that a greater proportion of tests resulted in negative result in the White British population (Additional file 1), suggesting that case finding and surveillance efforts were not disproportionately focused within the South Asian population. Secondly, there is likely to be substantial variation in testing capacity throughout the initial phase as services become overwhelmed and adapt to demand. This could lead to variation in the mix of self reported cases and those found through active case finding. There is no clear way to evaluate this in the present case as the source of cases is not recorded. This could impact perceived inequality in risk as actively found cases from known outbreaks could be favoured over self-reported cases and vice versa depending on the priorities of the local response.

Furthermore, the accuracy of ethnicity assignment using Onomap must play a role in the interpretation of these results [22]. Since previous verification studies show that Onomap has higher sensitivity when assigning British ethnicity compared to non-British ethnicities and lower specificity assigning British comparted to others (Additional file 1), it is likely that non-British ethnicities were underrepresented in the assigned data and British ethnicity was over represented. In particular, Black ethnicities may be highly under-represented, with a sensitivity of only a quarter (Additional file 1) [22], compared to other ethnicities which had a sensitivity of over three-quarters. Other Black ethnicities were not evaluated by Lakha et al. This poor performance suggests little should be interpreted from the proportion of cases Black ethnicities represent. However, this is not relevant for this analysis since the major focus is on South Asian and White British ethnicities. When considering this comparison explicitly, the performance in Lakha et al. suggests that South Asians are more likely to be under represented than over represented in the assigned data, suggesting that estimates for relative risk are conservative. In contrast British ethnicity is likely to be over-estimated, suggesting that disparities may have been even greater than identified in this analysis. Since these potential biases are not expected to vary over time, our analysis of variation in disparities over time should not have been further affected.

## Conclusions

This detailed analysis of the early phase of the UK pH1N1 epidemic in 2009 indicates that there may be a connection between the initiation of sustained transmission with the introduction of infection to more deprived areas and South Asian populations, both in Birmingham and London. This phenomenon resulted in higher risk of infection in the most deprived areas and South Asians during the containment phase of the epidemic, particularly in Birmingham and particularly in children under the age of 19 years. The combination of higher incidence in children and more pronounced disparities by deprivation status suggest that children are an important factor in driving inequalities in inluenza transmission in these settings.

## Supplementary Information

**Additional file 1:** Supplementary information for: Changing socio-economic and ethnic distribution of cases over the containment phase of the UK Influenza A H1N1 epidemic in 2009 – a comparison of London and Birmingham.

## Data Availability

The data that support the findings of this study are available from Public Health England but restrictions apply to the availability of these data, which were used under license for the current study, and so are not publicly available. The data can be requested directly from Public Health England.

## Abbreviations

UK: United Kingdom
pH1N1: Influenza A(H1N1)pdm09
NPFS: National Pandemic Flu Service
ONS: Office for National Statistics
LSOA: Lower super output area
PHE: Public Health England
IMD: Index of multiple deprivation
CI: Confidence interval
RR: Relative risk
NHS: National Health Service
