# Supplementary material for: Changing socio-economic and ethnic disparities in influenza/A/H1N1 infection early in the 2009 UK epidemic: a descriptive analysis
Source: BMC Infect Dis. 2021 Dec 11;21:1243. doi: 10.1186/s12879-021-06936-5 (PMC8665325; doi:10.1186/s12879-021-06936-5)
Supplement: Supplementary file 1 — Additional file 1: Supplementary information for: Changing socio-economic and ethnic distribution of cases over the containment phase of the UK Influenza A H1N1 epidemic in 2009 – a comparison of London and Birmingham. [file 12879_2021_6936_MOESM1_ESM.docx]

Supplementary information for: Changing socio-economic and ethnic distribution of cases over the containment phase of the UK Influenza A H1N1 epidemic in 2009 – a comparison of London and Birmingham

**Details of the Onomap software and verification**

Onomap version 2 (used for the analysis described in this paper) was developed in 2009 [1]; this version of the software consists of a database of names derived from public registries of over 26 countries and includes 448,657 surnames and 253,881 forenames [2]. The names are each classified by cultural ethnic and linguistic group by evaluating the community structure of the name network (linked by fore- and surnames). The lowest level classification is “Onomap type” which occurs as a community within the name network, along with this type is a probability score which gives likelihood of a name-type match, which is calculated from the share of the population with the particular name that can be assigned to that type in the training dataset (2001 census). As I stated before, Onomap software assesses both fore- and surname to assign an ethnicity classification. If the assignment conflicts, the software assigns the name-type match with the highest probability score.

Evaluation of the performance of the software has been undertaken by Lakha et. al. [2] in a study which tested the software’s performance against multiple datasets in Scotland by comparing assigned ethnicity with parents’ country of birth. The Sensitivity, Specificity, positive predictive value and Negative predictive value were reported for each ethnic classification. For clarity I present summary results here in Table S1. Notably, the assignment of ethnicity had higher specificity than sensitivity for all ethnicities except British. Complementarily, all non-British ethnicities were assigned with near perfect specificity, whereas British births had relatively poor specificity (68%). I include implications for this variation in sensitivity and specificity in the discussion of this chapter. African ethnicities were assigned with poor sensitivity.

Table S1 Comparison of Onomap results against general register office for Scotland (GROS) birth registration (males and females together). PPV, positive predictive value; NPV, negative predictive value; CI, confidence interval. [Directly from Table 1 in Lakha et. al [2]]

**Confidence intervals**

Confidence intervals of proportions were calculated as:

$$p\pm1.96\sqrt{\frac{p\left( 1-p \right)}{n}}$$

Where p is the proportion and n is the total population from which the proportion has been drawn.

Confidence intervals of all relative risks were calculated as:

$$\exp\left( LN(RR)\pm1.96\sqrt{\frac{{(n}_{pop}-x_{pop})/x_{pop}}{n_{pop}}+\frac{{(n}_{cases}-x_{cases}){/x}_{cases}}{n_{cases}}} \right)$$

Where n_pop_ is the total population and n_cases_ is the total number of cases and x_pop_ is the population in the particular sub-group and x_cases_ is the number of cases in the particular sub-group.

**Missing data**

I evaluated the proportion of reported cases with a missing postcode by date collected, age, gender and case status:

I ran a logistic regression for missing postcode with explanatory variables: Age, date recorded and whether tests were carried out.

Missing postcodes per onset date


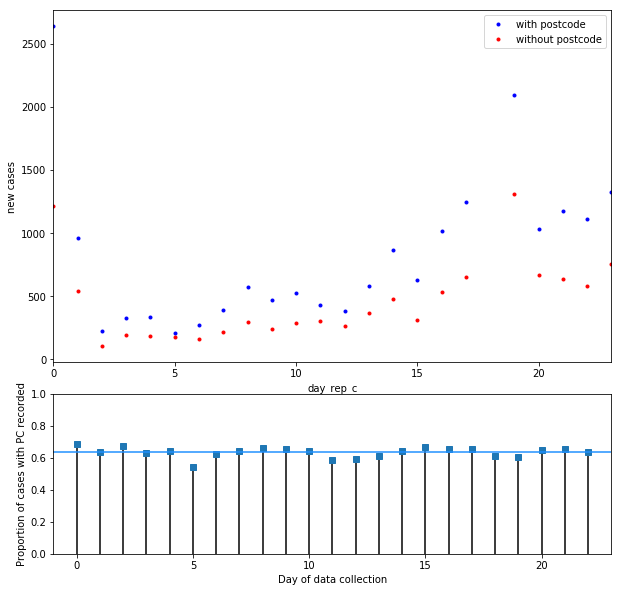


Figure S1 Missing postcodes per onset date

Missing postcodes per age group


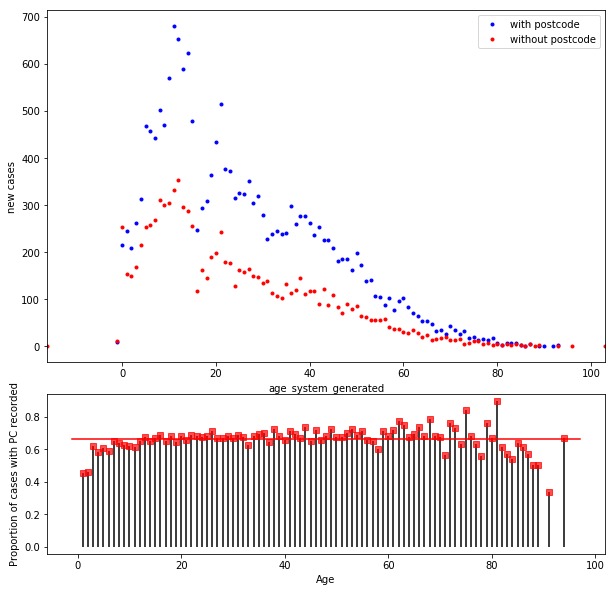


Figure S2 Missing postcodes per age group

**Missing postcodes by test status**


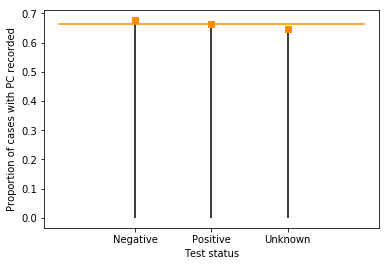


Figure S3 Missing postcodes by test status

**Missing postcodes by gender**


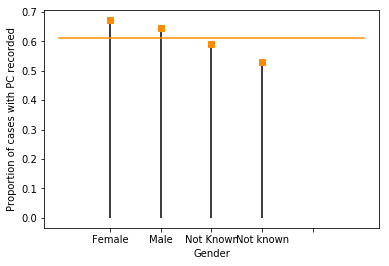


Figure S4 Missing postcodes by gender

**Logistic regression for predictors of missing post code:**

I performed a logistic regression to identify predictors of a missing postcode in the data.

Table S2A Results from Logistic Regression

| **Dep. Variable:** | PC_pres | **No. Observations:** | 28428 |
| --- | --- | --- | --- |
| **Model:** | Logit | **Df Residuals:** | 28424 |
| **Method:** | MLE | **Df Model:** | 3 |
| **Date:** | Wed, 08 Nov 2017 | **Pseudo R-squ.:** | 0.004056 |
| **Time:** | 17:21:05 | **Log-Likelihood:** | -18108. |
| **converged:** | True | **LL-Null:** | -18182. |
|  |  | **LLR p-value:** | 9.091e-32 |

Table S2B Results from Logistic Regression

|  | **coef** | **std err** | **z** | **P>\|z\|** | **[0.025** | **0.975]** |
| --- | --- | --- | --- | --- | --- | --- |
| **Intercept** | 0.4115 | 0.037 | 10.985 | 0.000 | 0.338 | 0.485 |
| **age_system_generated** | 0.0062 | 0.001 | 8.315 | 0.000 | 0.005 | 0.008 |
| **day_rep_c** | -0.0010 | 0.002 | -0.619 | 0.536 | -0.004 | 0.002 |
| **tested** | 0.2105 | 0.026 | 8.122 | 0.000 | 0.160 | 0.261 |

**2 Percentage of cases tested and tests positive by ethnic group**


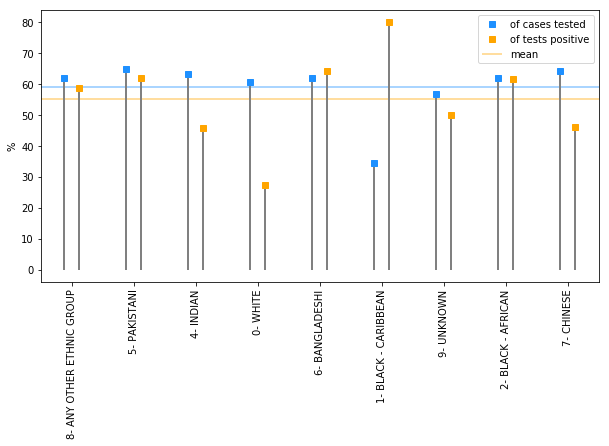


Figure S5 Percentage of cases tested and tests positive by ethnic group

- The proportion of tests that were positive is much lower in the white population. This could mean that there was an inflated reporting rate in this population.
- Noticeably, the test rate of cases was consistent for most ethnicities (around 60-65%) but this drops to less than 40% in black Caribbean’s. There were very small number of cases though - so may not be significant.

**3 Additional results**

**
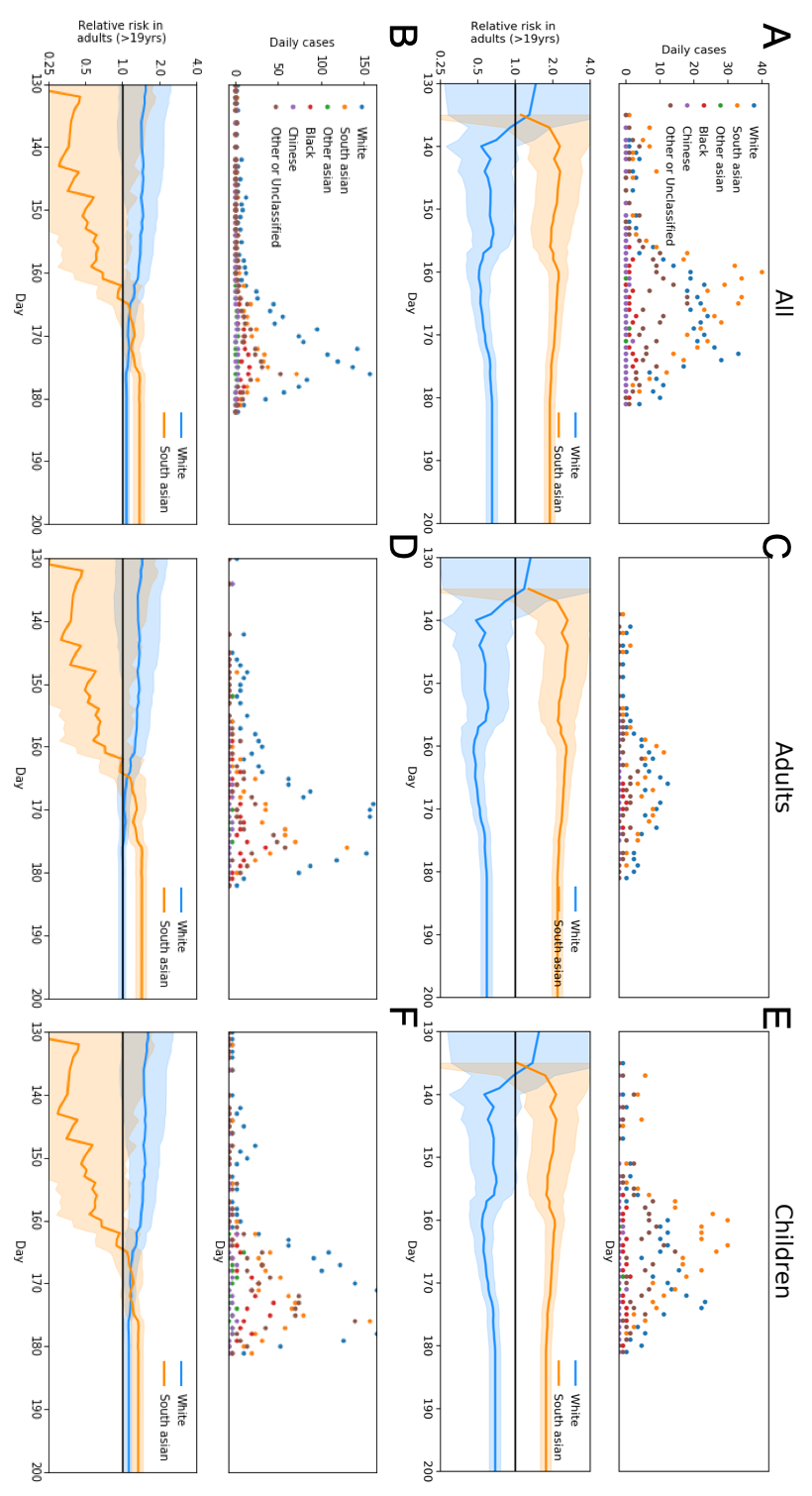
**

Figure S6 Ethnic breakdown of cases. i) Daily incidence in each ethnic group identified by Onomap, ii) Relative risk in White (Blue) and South Asian (Orange) populations for A) Birmingham , B) London, C) Adults in Birmingham (>19), D) Adults in London (>19), E) Children in Birmingham (<=19) and F) Children in London (<=19).

References

1. Mateos P, Longley PA, O’Sullivan D. Ethnicity and Population Structure in Personal Naming Networks. O’Rourke D, editor. PLoS One. 2011;6: e22943. doi:10.1371/journal.pone.0022943

2. Lakha F, Gorman DR, Mateos P. Name analysis to classify populations by ethnicity in public health: Validation of Onomap in Scotland. Public Health. 2011;125: 688–696. doi:10.1016/J.PUHE.2011.05.003
